# Anhydroicaritin suppresses tumor progression via the PI3K/AKT signaling pathway in hepatocellular carcinoma

**DOI:** 10.18632/aging.204948

**Published:** 2023-08-08

**Authors:** Houhong Wang, Wenli Chen, Yayun Cui, Huihui Gong, Amao Tang

**Affiliations:** 1 Department of General Surgery, The Affiliated Bozhou Hospital of Anhui Medical University, Bozhou 236800, Anhui, China; 2 Department of Cancer Radiotherapy, The First Affiliated Hospital of USTC, Division of Life Sciences and Medicine, University of Science and Technology of China (Anhui Provincial Cancer Hospital), Hefei 230031, Anhui, China; 3 Faculty of Health and Life Sciences, Oxford Brookes University, Oxford OX3 0BP, United Kingdom; 4 Department of Gastroenterology, The Affiliated Hangzhou First People’s Hospital, Zhejiang University School of Medicine, Hangzhou, Zhejiang, China

**Keywords:** Bushen Huayu Decoction, Anhydroicaritin, hepatocellular carcinoma, molecular docking, experimental validation

## Abstract

Background: Hepatocellular carcinoma (HCC) is one of the most malignant tumors. The *in vitro* experiments on the application of Anhydroicaritin (AHI), the active ingredient of Bushen Huayu Decoction, in HCC treatment remain limited, particularly regarding its molecular mechanism.

Methods: The TCMSP platform was used for drug ingredient screening. The GeneCards database and DisGeNET database are used to collect liver cancer targets. PPI network construction of active component-target intersection target was completed with string database. The GO and KEGG pathway analyses were performed via bioinformatics analysis. The molecular docking was used to confirm AHI’s target proteins. The *in vitro* experiments were performed to validate the effect of AHI on HCC cell and explore the molecular mechanism by western blotting analysis.

Results: Through the intersection, 155 intersection targets are finally obtained. The top 15 active ingredients were quercetin, kaempferol, beta-sitosterol, luteolin, beta-carotene, Stigmasterol, naringenin, formononetin, baicalein, Anhydroicaritin, isorhamnetin, licochalcone, 7-O-methylisomucronulatol, aloe-emodin and 8-O-Methylreyusi. The molecular mocking analysis showed that the four active components (quercetin, kaempferol, luteolin and AHI) and targets had a good binding activity (affinity ≤ 5 kcal/mol). *In vitro* experiments reveled that AHI could suppress tumor proliferation, invasion and metastasis of HCC cells. Further analysis showed that AHI inhibited tumor growth by PI3K/AKT signal pathway in HCC.

Conclusions: The Bushen Huayu Decoction and its active ingredient AHI could fight HCC. The potential mechanism may be associated with inhibiting the activation of PI3K/AKT signal pathway, which may serve as a potential treatment for HCC therapy.

## INTRODUCTION

Hepatocellular carcinoma (HCC) is one of the most malignant tumors. At present, the cancer-related mortality rate is the second in the world, second only to lung cancer [1–3]. The long-term survival rate of HCC patients is relatively low, and the 5-year survival rate is less than 20%, in which cancer cell metastasis is the main cause of death [4]. Traditional Chinese medicine (TCM) is widely recognized for its multi-target and synergistic effect on HCC [5–7]. More and more evidences show that TCM can inhibit the cell malignancy in HCC and the progress of epithelial-mesenchymal transition (EMT) [6, 8]. Huang et al. demonstrated that Huanglian Jiedu decoction, one of the most commonly used classic TCM formulae, could remarkably suppress HCC cells growth *in vitro* and *vivo* [9]. Wu et al. showed that Yiqi Jianpi Jiedu formula exerts its anti-HCC effect of HCC by β-catenin, mitogen-activated protein kinase 3 (MAPK3), PI3K/AKT pathway and ras homolog family member A (RHOA) [10]. Liu et al. demonstrated that astragalus membranaceus directly down-regulated MT1G through daidzein to promote ferroptosis of HCC cells and improved prognosis for HCC [11].

The active ingredients of Bushen Yangxue Huayu Decoction included Radix Rehmanniae, Eucommia ulmoides, Radix Paeoniae Alba, Radix Achyranthis, Radix Astragali, Herba Epimedii, Radix Angelicae Sinensis, Radix Carthamus, Caulis spatholobi, Cistanche deserticola, Rhizoma dogleg, and Radix Aucklandiae. Icariin (ICA) is the main active ingredient of Epimedium [12]. ICA, as a traditional Chinese medicine for tonifying the kidney and strengthening yang, is widely used in the prevention and treatment of bone loss related to breast cancer patients, as well as in the treatment of perimenopausal syndrome [13–15]. It can improve symptoms such as hot flushes, sweating and irritability in breast cancer patients with chemotherapy amenorrhoea. Patents have shown that icariin and epimedium flavonoids containing icariin can treat diseases associated with myelin lesions [16]. The main active components of icariin include ICA, icariin (ICT), etc. Icariin does not have significant estrogen-like effect, and the hydrolysates of icariin, icariin and Anhydroicaritin (AHI), may be the real pharmacological active components. AHI is a flavonoid compound with anti-tumor biological and pharmacological effects [17]. After oral administration, it can be decomposed by intestinal bacteria to produce AHI and other metabolites [18]. A previous study revealed that AHI suppressed EMT by increasing glutathione peroxidase 1 in breast cancer cell [19]. In addition, AHI was reported to play an anti-breast cancer role by inhibiting cancer cell proliferation and metastasis [20]. AHI plays a promising role in breast cancer, however, studies on the application of AHI in HCC treatment remain limited, especially at the molecular mechanism.

In the present study, the anti-hepatoma mechanism of AHI was deeply studied through bioinformatics analysis, network pharmacology, molecular docking and *in vitro* experiments. Network pharmacology and colony formation experiments demonstrated the anti-HCC potential of AHI. Western blot analysis confirmed that AHI suppressed tumor progression via the PI3K/AKT signaling pathway in HCC.

## RESULTS

### Target integration of Bushen Huoxue Huayu Decoction

Through TCMSP platform and Chinese herbal medicine platform, OB ≥ 30%, DL ≥ 0 18 was acted as the filter condition. The active components of Radix Rehmanniae, Eucommia ulmoides, Radix Paeoniae Alba, Achyranthes bidentata, Radix Astragali, Epimedium, Angelica sinensis, safflower, Caulis spatholobi, Cistanche deserticola, Rhizoma dogleg and Radix Aucklandiae were retrieved respectively. There were 145 active components in the soup of invigorating the kidney, activating blood circulation and removing stasis, and a total of 3944 targets were collected. After deleting the duplicates, 273 target genes were obtained by gene name transformation of the target protein of the active ingredient using Uniprot database.

### Liver cancer target integration

**Figure 1:**
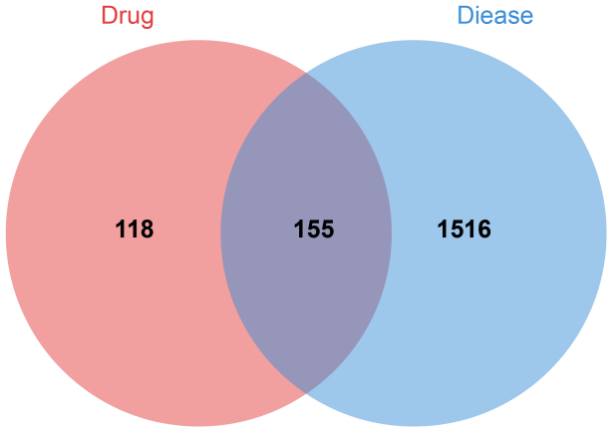
Venn diagram of drug-disease intersection target.

We searched with “multiple myeloma” as the key word, collected the relevant targets of MM, summarized the results of the disease database, and finally obtained 1671 disease genes. The disease target and the active ingredient target were intersected to obtain 155 intersection targets (Figure 1).

### Active component-target network analysis

**Figure 2:**
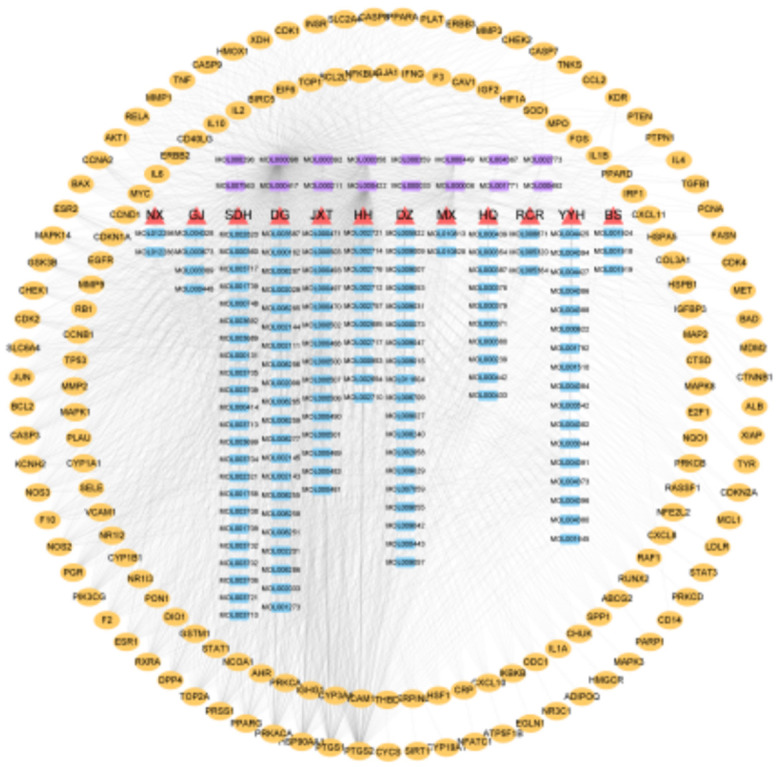
**Active component-target network.** **▲** Abbreviation of the name of traditional Chinese medicine, MX: Muxiang and so on. **■** the active ingredient (purple is the common active ingredient); **●** Target gene.

Cytascape was used to draw the “active component-target” network diagram of 155 intersection targets (Figure 2). Network Analyzer was used to calculate and analyze 312 nodes, 2154 edges, and the average degree value was 13.8. The top 15 active ingredients were screened according to the degree value, as shown in Table 1.

**Table 1:** Active ingredients with the top 15 degrees.

**Mol ID**	**English name**	**Degree**	**Chinese medicine**
MOL000098	quercetin	675	Eucommia ulmoides, achyranthes bidentata, astragalus, epimedium, safflower, cistanche deserticola
MOL000422	kaempferol	252	Eucommia ulmoides, white peony, astragalus, epimedium, safflower, dog ridge
MOL000358	beta-sitosterol	110	Eucommia ulmoides, white peony, achyranthes bidentata, angelica sinensis, safflower, caulis spatholobi, cistanche deserticola
MOL000006	luteolin	101	Epimedium, safflower, caulis spatholobi
MOL002773	beta-carotene	38	Eucommia ulmoides, safflower
MOL000449	Stigmasterol	37	Rehmannia glutinosa, angelica, safflower, caulis spatholobi, woody incense
MOL004328	naringenin	27	Dog ridge
MOL000392	formononetin	26	Astragalus, Caulis spatholobi
MOL002714	baicalein	26	safflower
MOL004373	Anhydroicaritin	24	Epimedium
MOL000354	isorhamnetin	24	Astragalus membranaceus
MOL000497	licochalcone a	22	Caulis spatholobi
MOL000378	7-O-methylisomucronulatol	22	Astragalus membranaceus
MOL000471	aloe-emodin	21	Caulis spatholobi
MOL000468	8-o-Methylreyusi	20	Caulis spatholobi

### PPI analysis of drug active components-hepatoma intersection target

**Figure 3:**
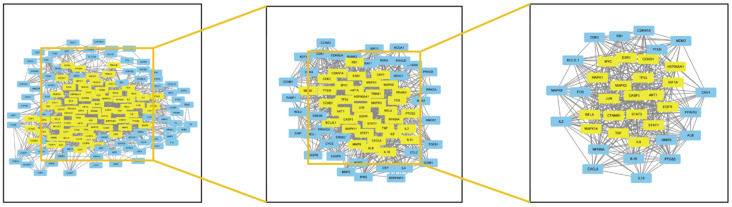
Analysis diagram of intersection target network.

**Figure 4:**
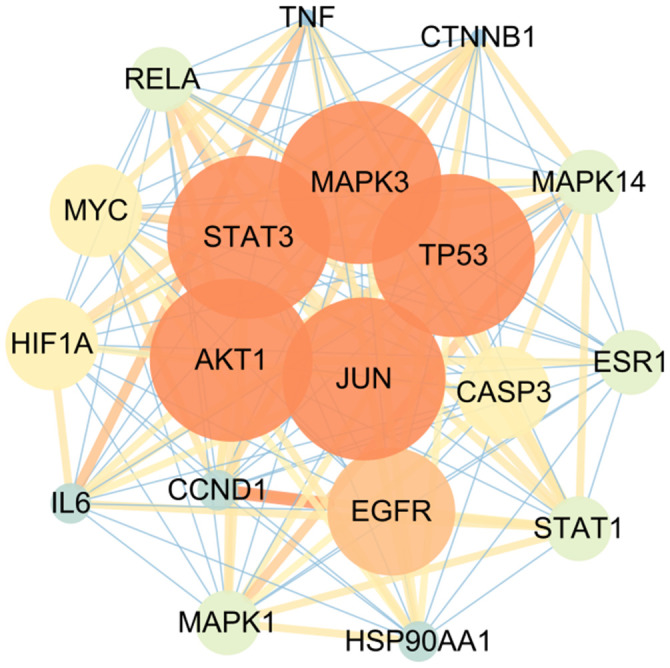
Core network diagram of intersection target.

Next, we imported 155 intersection targets into the string database and adjusted the minimum required interaction score to high confidence (0.7) to obtain protein interaction network and corresponding network data. The data were imported into Cytoscape for analysis, and 148 nodes and 1490 edges are obtained, with an average degree of 12.79. The CytoNCA plug-in was used for protein clustering of gene groups, and the centrality analysis was carried out by Betweenness (BC), Closeness (CC) and Degree (DC) analysis conditions. TP53, AKT1, STAT3, JUN and MAPK3 are the core targets in the network, as shown in Figures 3, 4, and the top 15 targets in the core network are shown in Table 2.

**Table 2:** The top 15 targets in the core network.

**Targets**	**Degree**	**Targets**	**Degree**	**Targets**	**Degree**
TP53	18	EGFR	17	ESR1	15
AKT1	18	CASP3	16	STAT1	15
STAT3	18	MYC	16	RELA	15
JUN	18	HIF1A	16	MAPK14	15
MAPK3	18	MAPK1	15	IL6	14

### GO enrichment analysis

**Figure 5:**
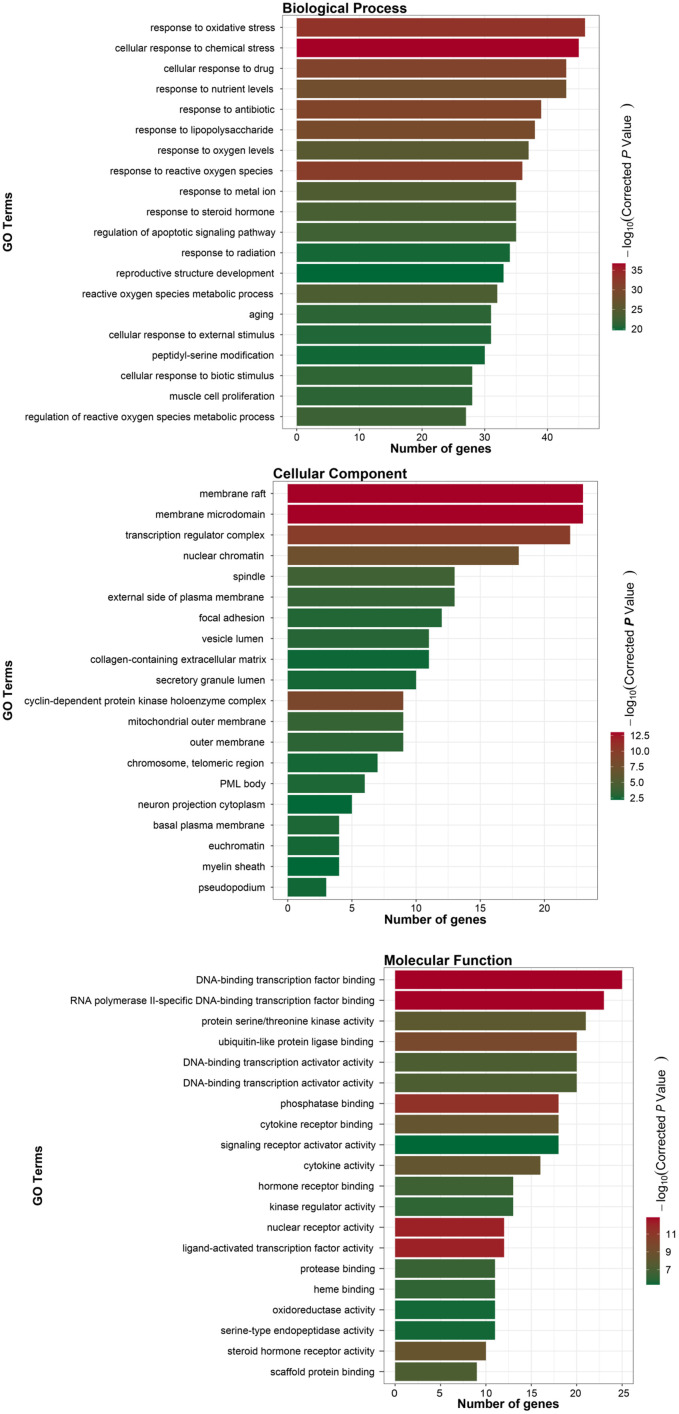
GO analysis of intersection target.

The R package was used for GO enrichment analysis of the intersection targets. A total of 629 biological process (BP) entries were obtained, involving cell response to chemical stress and oxidative stress, response to oxygen level and metal ions, regulation of active oxygen metabolism, regulation of apoptosis signal pathway, proliferation of muscle cells, aging and other processes. A total of 36 cell component (CC) entries involved transcriptional regulatory complex, nuclear chromatin, cyclin kinase complex, mitochondrial outer membrane, cytoplasmic membrane and other components and 80 molecular function (MF) entries, involving RNA polymerase II specific DNA-binding transcription factor binding, DNA-binding transcription factor binding, nuclear receptor activity, phosphatase binding, ubiquitin-like protein ligase binding, protein serine/threonine kinase activity and other functions. According to the number and diversity of genes, the top 20 items of three cluster analysis are drawn, as shown in Figure 5.

### KEGG pathway analysis

**Figure 6:**
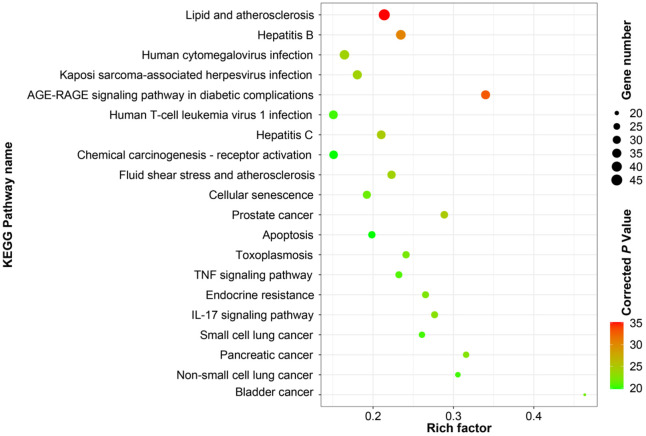
KEGG enrichment analysis of intersection target.

The KEGG enrichment analysis of the obtained intersection targets was carried out using R package. The results showed that there were 173 pathways with p value<0.05, and the results of the top 20 entries were sorted according to the corrected p value, as shown in Figure 6. These findings revealed that the genes were mainly enriched in AGE-RAGE signal pathway, IL-17 signal pathway, endocrine resistance, TNF signal pathway, cell aging, apoptosis, p53 signal pathway, PI3K-AKT signal pathway and other signal pathways.

### Molecular docking analysis

**Figure 7:**
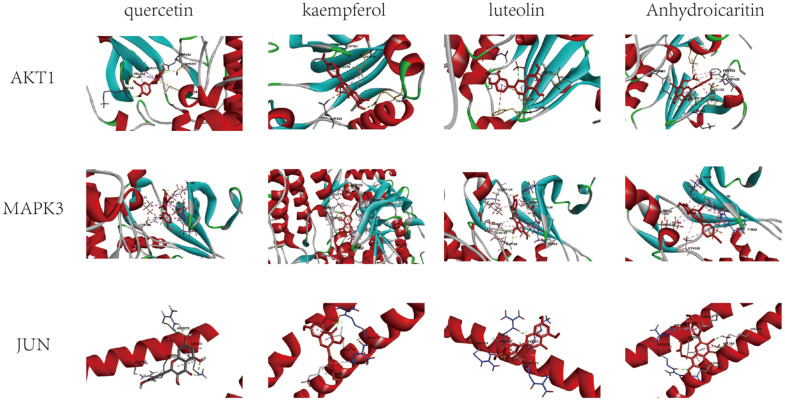
Molecular docking diagram of some proteins and active ingredients.

The four active components (quercetin, kaempferol, luteolin and Anhydroicartin) with high intermediate value in the core network of the intersection target were linked with the three core proteins (AKT1, MAPK3, JUN) in the PPI network by using Autodock software. The lower the binding energy of the two, the more stable the binding of ligand and receptor. The results show that the binding energy of the four active components and three core proteins is less than -5kcal·mol-1, as shown in Table 3. The results were imported into the Discovery studio software for visual optimization, and the three-dimensional molecular docking diagram was output (Figure 7).

**Table 3:** Binding energy of core active compound and core protein.

**Chemical compound**	**MOL ID**	**Affinity(kcal/mol)**		
		**AKT1**	**MAPK3**	**JUN**
Quercetin	MOL000098	-7.9	-9.1	-5.6
Kaempferol	MOL000422	-7.8	-9.4	-5.5
Luteolin	MOL000006	-8.7	-9.5	-5.9
Anhydroicaritin	MOL004373	-8.7	-9.6	-6

Quercetin forms hydrogen bonds with ASP292, LYS18 and THR291 in the amino acid residues of AKT1, and hydrophobic forces with MET281, ALA177 and VAL164. Kaempferol forms hydrogen bonds with GLU191 and GLY162 in the amino acid residues of AKT1, and generates hydrophobic forces with LYS179, LEU181 and ASP292. Luteolin forms hydrogen bonds with GLU198, GLY162, LYS179 and THR195 in the amino acid residues of AKT1, and generates hydrophobic forces with LEU181 and VAL164. Anhydroicartin forms hydrogen bonds with LYS159, ASP292 and ASP439 in the amino acid residues of AKT1, and hydrophobic forces with GLU234, LYS179, PHE442, LEU156, PHE438 and MET281.

### AHI inhibits colony formation, proliferation and migration of hepatocellular carcinoma cells

**Figure 8:**
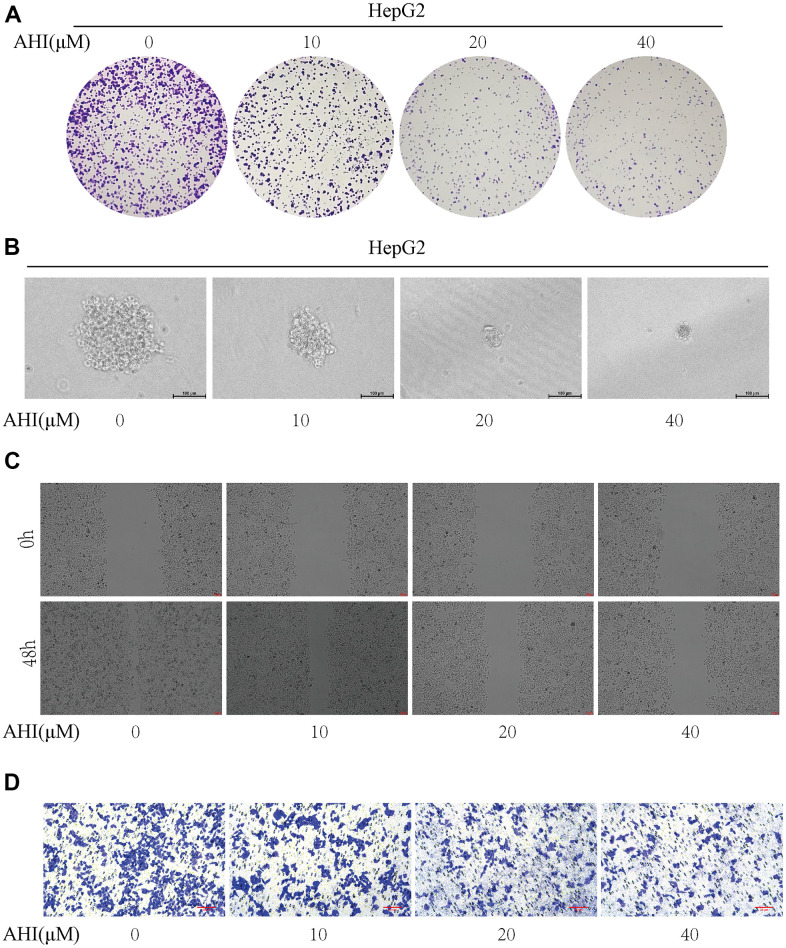
**AHI has potential anti-liver cancer properties.** (**A**) Colony formation of HepG2 after treatment with different concentrations of AHI. (**B**) Sphere formation of HepG2 after treatment with different concentrations of AHI. (**C**) Effects of AHI on the migration of HCC cell. (**D**) Effects of AHI on the invasion of HCC cell.

The HepG2 cell was used to validate the effects of AHI on cell growth. In the colony formation assay, HepG2 cells were treated with different concentrations of AHI for 7 days. The result showed that the number and size of cell clones decreased with the increase of AHI concentration (Figure 8A). Similarly, the results of tumor cell spheroidization assay revealed that the sphere-formation of HepG2 cells treated with AHI was obviously inhibited compared control group (Figure 8B). Then, we further validated the effects of AHI on liver cancer cells by cell migration and invasion assays. The migration activity of the HepG2 cells was significantly suppressed by AHI compared with control group (Figure 8C). Moreover, the invasion activities of HepG2 cells were significantly inhibited by AHI (Figure 8D). These results indicated that AHI can inhibit the migration and invasion activity of liver cancer cells. These findings suggested that AHI has potential anti-liver cancer properties.

### AHI suppressed PI3K/AKT pathway

**Figure 9:**
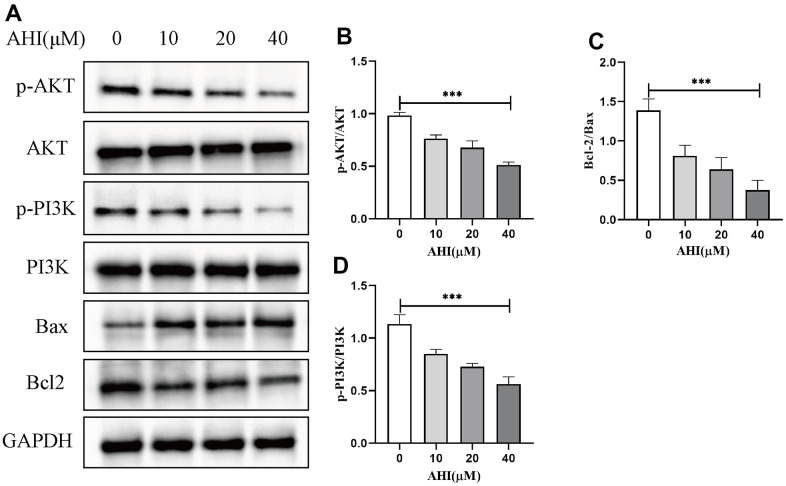
**The protein levels of Bax, Bcl2 and key molecules of PI3K/AKT pathway were detected by Western blotting.** (**A**) Effect of AHI on the expression of Bax, Bcl2 and PI3K/AKT signalling pathway proteins in HCC cells. (**B**) Ratio of p-AKT to AKT protein expression (***p<0.001). (**C**) Ratio of Bcl2 to Bax protein expression (***p<0.001). (**D**) Ratio of p-PI3K to PI3K protein expression (***p<0.001).

Western blot analysis was performed to compare the expression of Bax, Bcl2, p-PI3K, PI3K, p-AKT and AKT in HepG2 cells with AHI treatment. Bax/Bcl2 are key proteins related to apoptosis. As shown in Figure 9, AHI significantly decreased Bcl2 with a concomitant upregulation of Bax in HepG2 cells. To better understand the molecular mechanism of AHI on tumor growth and metastasis, we examined the expression levels of PI3K/AKT pathway. The results revealed that AHI can suppress the expression of p-PI3K and p-AKT (Figure 9), suggesting a novel mechanism of AHI in inhibiting the PI3K/AKT signaling pathway during liver cancer treatment.

## DISCUSSION

TCM resources are an important source of chemical entities supporting drug discovery [21]. There are about 10000 kinds of TCM resources [22]. Therefore, it is necessary to tap the huge potential of TCM. With the continuous development of modern science and technology, the development of TCM should also be combined with intelligent and efficient methods.

Liver cancer is one of the common malignant tumors, and the global incidence rate and mortality are increasing year by year [23]. Cancer cell metastasis is the main reason for poor prognosis and death of liver cancer. TCM plays an important role in the comprehensive treatment of liver cancer in China. The research shows that the TCM treatment characterized by holistic concept and treatment based on differentiation of symptoms and signs, with the concept of “supporting the right and removing evil” as the anti-liver cancer concept, can play an anti-tumor role through multi-level, multi-target and multi-channel [24–26]. TCM can play an anti-tumor role through multi-level, multi-target and multi-channel, thus prolonging the survival period of patients with liver cancer, improving clinical symptoms and improving quality of life [11, 27–29].

The active ingredients of Bushen Yangxue Huayu Decoction included Radix Rehmanniae, Eucommia ulmoides, Radix Paeoniae Alba, Radix Achyranthis, Radix Astragali, Herba Epimedii, Radix Angelicae Sinensis, Radix Carthamus, Caulis spatholobi, Cistanche deserticola, Rhizoma dogleg, and Radix Aucklandiae. The present study aimed to classify the molecular mechanism of Bushen Yangxue Huayu Decoction in treating HCC. A total of 145 core active ingredients were identified. Moreover, using Uniprot database, 273 target genes were obtained by gene name transformation of target protein of active ingredient. The results of active component-target network analysis showed that the top 15 active ingredients were quercetin, kaempferol, beta-sitosterol, luteolin, beta-carotene, Stigmasterol, naringenin, formononetin, baicalein, Anhydroicaritin, isorhamnetin, licochalcone a, 7-O-methylisomucronulatol, aloe-emodin and 8-O-Methylreyusi, respectively. In addition, PPI analysis of drug active components-hepatoma intersection target found that TP53, AKT1, STAT3, JUN and MAPK3 were the core targets in the network.

Next, the four active components (quercetin, kaempferol, luteolin and AHI) with high degree in the core network of the intersection target were linked with the three core proteins (AKT1, MAPK3, JUN) in the PPI network by using Autodock software. It was found that almost the four active components and targets had a good binding activity (affinity <- 5 kcal/mol). Anhydroicartin, a prenylated flavonoid naturally occurring in several species of Epimedium, is recognised as one of the active compounds in the famous traditional Chinese herb Epimedium. Anhydroicartin exhibits a variety of biological activities such as activating apoptosis and inhibiting the growth of cancer cells, protecting against beta-amyloid-induced neurotoxicity and promoting neuronal and cardiac cell differentiation [30, 31]. However, there are fewer studies on the role of AHI in HCC. We thus performed an experiment validation *in vitro* of active component, AHI. Our findings revealed that AHI treating was associated with repression of the abilities of proliferation, migration and invasion in HCC cells. Next, the changes in key proteins of the PI3K/AKT pathway were analyzed in detail. It was found that AHI could suppress the tumor growth, invasion by inhibiting PI3K/AKT signal pathway.

## CONCLUSIONS

Through the validation of bioinformatics and experimental research, this study analyzed the potential mechanism of Bushen Huayu Decoction and its active ingredient AHI against HCC. AHI plays an anti-HCC effect by suppressing tumor proliferation and metastasis. The potential mechanism may be associated with inhibiting the activation of PI3K/AKT signal pathway, which may serve as a potential treatment for HCC therapy, providing a new direction for the clinical management of HCC.

## MATERIALS AND METHODS

### Target collection of Bushen Yangxue Huayu Decoction

Through the TCMSP platform (https://tcmspw.com/tcmsp.php), the set conditions were oral bioavailability (OB) ≥ 30%, and drug-likeness (DL) ≥ 0.18 to screened out drug gradients. The active ingredients and corresponding targets of 12 traditional Chinese medicines, including Radix Rehmanniae, Eucommia ulmoides, Radix Paeoniae Alba, Radix Achyranthis, Radix Astragali, Herba Epimedii, Radix Angelicae Sinensis, Radix Carthamus, Caulis spatholobi, Cistanche deserticola, Rhizoma dogleg, and Radix Aucklandiae, were searched. Finally, the collected active ingredient targets will be converted into corresponding gene names and ID with the help of Uniprot database (https://www.uniprot.org).

### Liver cancer target collection

GeneCards database (https://www.genecards.org/) and DisGeNET database (https://www.Disgenet.org) were used for collecting disease targets. Then, we used “liver cancer” as the key word to search, filter and collect relevant targets, and filter by the median, and finally delete and merge the results of the two databases.

### Drawing of “active component-target” network

Next, we intersect the target of active ingredients of Bushen Huoxue Huayu Decoction and the target of liver cancer. The “active component-target” network is drawn by using Cytascape software, and the main active compounds are screened by using Network Analyzer according to the degree of calculation.

### Protein-protein interaction (PPI)

The string database (https://stringdb.org) was used to construct the PPI network for the active component-target intersection target, and the Cytoscape and CytoNCA plug-ins were used to analyze the centrality of the protein network to obtain the core protein with a higher degree.

### GO and KEGG enrichment analysis

Using the R language and ClusterProfiler, we used the modified Fisher exact test, and then set the cut-off value of p value corrected and corrected by the Benjamini-Hochberg multiple hypothesis test to 0.05, which was used to perform gene function annotation, cluster human genes as background, and default options and annotation categories. A significantly enriched KEGG pathway was identified using hypergeometric test and Benjamin Hochberg FDR correction.

### Molecular docking

The components quercetin, kaempferol, luteolin and Anhydroicartin were linked with the core proteins (AKT1, MAPK3, JUN) with higher degree in PPI network. The PubChem database (https://pubchem.ncbi.nlm.nih.gov/) was used to obtain the structure of the compound. The PDB database (https://www.rcsb.org/) was applied to get protein structure. Moreover, Autodock Vina software was used for molecular docking, and Discovery Studio software was used to optimize the output of the results.

### Cell line

The liver cancer cell line, HepG2, was purchased from Procell Life Science and Technology Co. Ltd. (Wuhan, China). The HepG2 cell was cultured in complete medium (RPMI 1640 plus 10% FBS and 1% penicillin - streptomycin). Cell was cultured in a 37° C incubator with a humidified 5% CO_2_ atmosphere.

### Colony formation assays

Anhydroicaritin (AHI) was purchased from Shanghai Weiao Medical Technology Co. Ltd. (Shanghai, China). Colony formation assays were used to further validate the inhibitory effect of AHI on the tumorigenicity of liver cancer cell. A total of 2000 HepG2 cells/well were seeded in 6-well plates. Then, the different concentrations of AHI were performed to treat cells for 7 days. After fixing with 5% methanol for 30 minutes, they were dyed with crystal violet solution for 10 minutes, and counted the colonies containing more than 50 single cells under the microscope.

### Tumor cell spheroidization assays

HepG2 cells were digested with trypsin and made into single-cell suspension. After washing with PBS, they were inoculated into serum-free DMEM/F12 medium containing basic fibroblast growth factor (bFGF), epidermal growth factor (EGF) and leukaemia inhibitory factor (LIF) and other factors, and then cultured in 25cm^2^ low-adhesion culture flask. 10ml of the above medium was added to regulate the number of cells to 1 × 10^5^. They were incubated in a constant temperature of 37° C and 5% CO_2_ incubator, shaken gently once every 24 hours, and changed the liquid by half volume every 3 days. After two weeks, we can basically see the globular cells.

### Wound healing and transwell assays

HepG2 cell treated with different concentrations of AHI for wound healing assay was cultured in 6-well plates and grown to full confluence. Next day, we made artificial homogeneous wounds using a 200 μl pipette tip. Cells were serum-starved overnight after washing twice with PBS to remove debris. At 0 h and 48 h in the cell incubator, artificial scratch wound areas were photographed under microscopy. Migrate rate was calculated as follows: migrate rate % = (original scratch area−scratch area at 24h)/original scratch area×100%. Transwell assay was performed to assess cell migration. 8.0-μm transwell chambers in 24-well plates were used. A total of 10× 10^4^ cells suspended in 150 μl FBS-free medium were seeded onto top chambers for the migration assay, and 700 μl RPMI-1640 medium with 20% FBS was added to the lower chambers. After 36 h of incubation, cells that had invaded were fixed in 4% paraformaldehyde and stained with 0.1% crystal violet for counting. All assays were repeated three times.

### Western blotting (WB) analysis

Total proteins of HepG2 cell were extracted with RIPA buffer which added protease inhibitors and phosphatase inhibitors (200:1:1), and then was quantified by BCA Assays. The lysates were separated on SDS-PAGE gels under constant voltage and transferred onto 0.22μm polyvinylidene fluoride (PVDF) membranes. 5% non-fat dry milk was applied for blocking the blots for 2 h at room temperature. And the blots were incubated with primary antibodies at 4° C overnight. Next day, HRP-conjugated secondary antibodies were incubated 1 h at room temperature after washing with TBST for three times. All blots were examined by ECL reagent and visualized using a Tanon system. β-actin expression was used to quantify the data. The assay was repeated three times.

### Statistical analysis

Each group of experiments was repeated three times and data were analysed using GraphPad Prism 8.0 and SPSS 10.0, with results expressed as mean ± standard deviation $\left(\bar{\text{x}}\pm \text{s}\right)$, with differences considered statistically significant at p<0.05.

### Data availability

All supporting experiment data are available from the corresponding author upon request.
